# Circ_0001955 facilitates hepatocellular carcinoma (HCC) tumorigenesis by sponging miR-516a-5p to release TRAF6 and MAPK11

**DOI:** 10.1038/s41419-019-2176-y

**Published:** 2019-12-10

**Authors:** Zhicheng Yao, Ruiyun Xu, Lin Yuan, Mingxing Xu, Haiyun Zhuang, Yanjie Li, Yi Zhang, Nan Lin

**Affiliations:** 1 Department of General Surgery, The Third Affiliated Hospital, Sun Yat-sen University, Guangzhou, 510630 Guangdong Province China; 2 Department of Hepatobiliary Surgery, The Third Affiliated Hospital, Sun Yat-sen University, Guangzhou, 510630 Guangdong Province China

**Keywords:** Oncogenes, Cancer epidemiology

## Abstract

Circular RNAs (circRNAs) have been increasingly demonstrated to function as novel promising therapeutic RNA molecules for diverse human diseases, including cancer. Although the important role of circRNAs has been well documented in HCC, the complex mechanisms of circRNAs in HCC need to be elucidated. Here, a novel circRNA circ_0001955 was identified from three GSE datasets (GSE7852, GSE94508, and GSE97322) as a differentially expressed circRNA between HCC and normal samples. We revealed that circ_0001955, TRAF6 and MAPK11 levels were increased, while miR-516a-5p levels were decreased in HCC tumor tissues compared to adjacent normal tissues. Knockdown of circ_0001955 repressed HCC tumor growth in vitro and in vivo, while overexpression of circ_0001955 exhibited the opposite effect. Circ_0001955 was identified as a sponge for miR-145-5p and miR-516a-5p, and TRAF6 and MAPK11 were demonstrated to be two target genes of miR-516a-5p. In conclusion, circ_0001955 facilitated HCC tumorigenesis by sponging miR-516a-5p to release TRAF6 and MAPK11 expression.

## Introduction

Hepatocellular carcinoma (HCC) is the most frequently occurring malignancy in the liver worldwide^1,2^. The global incidence and mortality of HCC is still increasing in the majority of countries^3^. In China, HCC is ranked as the fourth most common tumor, accounting for more than 90% of all primary liver malignancies^4^. In the United States, HCC caused approximately 11,073 deaths in 2016 alone, which is nearly twice the number from decades ago^5^. In general, multiple effective therapeutic options are available to HCC patients who are diagnosed at an early stage, including surgical removal, liver transplantation and chemotherapy^6^. However, the prognosis of advanced HCC patients with metastasis remains dismal, even if multikinase inhibitors, such as sorafenib and regorafenib, are adopted^2,7^. Unfortunately, due to the lack of available screening agents and no obvious early symptoms, a considerable proportion of HCC patients are diagnosed at an advanced stage^8^. The effects of most therapeutic agents for advanced HCC patients are largely limited by the acquired drug resistance of HCC cells^9^. Even after surgical resection, the five-year survival rate of HCC patients with stage III is as low as 16%^10^. Investigating the pathogenesis of HCC might contribute to the identification of effective molecules for HCC diagnosis and treatment.

Circular RNAs (circRNAs) are a novel subtype of noncoding RNAs (ncRNAs) that widely exist in almost all subcellular compartments of eukaryotic cells^11,12^. In general, circRNAs are derived from exons in a back-splicing or lariat-circulating manner; however, in rare circumstances, circRNAs can also be derived from introns and untranslated regions^13^. In addition, circRNAs have been identified to bind with RNA-binding proteins^14^. Moreover, circRNAs can undergo translation^15,16^. Evidence has also shown that circRNAs are predominantly localized in the cytoplasm, where they can interact with microRNAs (miRNAs), another important subtype of ncRNAs, to regulate the target genes of miRNAs, thereby participating in the regulation of diverse biological processes including human cancers^17^. Recently, an increasing number of circRNAs have been demonstrated to play a role in the tumorigenesis of HCC through diverse mechanisms, revealing the complexity of HCC pathogenesis^13,18^. Nevertheless, the regulatory network of circRNAs in HCC needs further study.

To better understand the regulatory network of circRNAs in HCC, we analyzed the data of three GSE datasets (GSE7852, GSE94508 and GSE97322) downloaded from the Gene Expression Omnibus database (GEO, http://www.ncbi.nlm.nih.gov/geo) using the GEO2R online tool. Circ_0001955 was identified as one of the two differentially expressed circRNAs that were detected in all three GSE datasets. In this study, we aimed to investigate the biological roles of circ_0001955 in HCC tumorigenesis and attempted to explore the underlying regulatory mechanisms of circ_0001955 in HCC.

## Materials and methods

### Tissue specimens and cell culture

Tissue specimens were obtained from patients who were diagnosed with HCC between 2015 and 2019 in the Department of The Third Affiliated Hospital, Sun Yat-sen University. Every participant involved in this study provided written informed consent that was approved by the Ethics Committee of The Third Affiliated Hospital, Sun Yat-sen University. Collected tissue samples were frozen at −80 °C until use. A normal hepatic cell lines (LO2) and HCC cell lines (Huh-7, HepG2, SMMC-7721, Bel-7402, and Hep-3B) were all purchased from ATCC and maintained at 37 °C in DMEM culture medium containing 10% FBS.

### CircRNA expression profile analysis

Three HCC gene expression datasets were downloaded from the Gene Expression Omnibus database (GEO, http://www.ncbi.nlm.nih.gov/geo), and analyzed using the online software GEO2R (http://www.ncbi.nlm.nih.gov/geo/geo2r/) to screen for differentially expressed circRNAs.

### RT-PCR assay

Total RNA was extracted from HCC cells or tissues using TRIzol (Invitrogen, USA). After examining the quality of RNAs, 3 μg RNA was utilized as the template to synthesize cDNA using a Bestar qPCR RT kit (DBI Bioscience, China) following the manufacturer’s instructions. qRT-PCR was conducted with a 7500 Fast Real-Time PCR system (Applied Biosystems, USA) using a Bestar qPCR MasterMix kit (DBI Bioscience). U6 and GAPDH were applied as controls for miRNA and mRNA expression analysis, respectively. Data were calculated via the 2^−ΔΔCt^ method. The primers designed for this study are shown in Table 1.

**Table 1:** Primer sequences in RT-PCR assay

Gene	Sequence or target sequence
Circ_0001955-F	5′-GGTGCATCTGCAATAACTCG-3′
Circ_0001955-R	5′-ATTTCCCACATGGTCCAAAG-3′
GAPDH-F	5′-CACCCACTCCTCCACCTTTG-3′
GAPDH-R	5′-CCACCACCCTGTTGCTGTAG-3′
TRAF6-F	5′-ATGGCTTGCARYGACATGGAGAAG-3′
TRAF6-R	5′-TCAAAGTGAAGGTTCTGGGCCCCGAG-3′
MAPK11-F	5′-TGGCACCCATGAAATTGAGCAGTG-3′
MAPK11-R	5′-AGGGTTACAGACACATCCGTGCAT-3′
miR-516a-5p-F	5′-CTCAACTGGTGTCGTGGAGTCGGCAATTCAGTTGAGGAAAGTGC-3′
miR-516a-5p-R	5′-ACACTCCAGCTGGGTTCTCGAGGAAAGAAGC-3′
miR-145-5p-F	5′-AGTCCAGTTTTCCCAGGAATCCCT-3′
miR-145-5p-R	5′-GCTG TCAACGATACGCTACGT-3′
U6-F	5′-CTCGCTTCGGCAGCACA-3′
U6-R	5′-AACGCTTCACGAATT TGCGT-3′

### Oligonucleotide transfection

Circ_0001955 siRNAs (si-circ_0001955#1 and si-circ_0001955#2), si-NC, Lv-circ_0001955, Lv-NC, miR-NC, miR-516a-5p, miR-516a-5p mut, anti-miR-NC, anti-miR-516a-5p, and anti-miR-516a-5p mut were all obtained from Integrated Biotech Solutions (Shanghai, China). The wild-type or mutant miR-516a-5p binding sequence of circ_0001955 and the 3’-UTR of TRAF6 or MAPK11 were inserted into the psi-CHECK-2 plasmid to construct the luciferase reporter plasmids (Luc-circ_0001955, Luc-circ_0001955 mut, Luc-TRAF6, and Luc-MAPK11). Lipofectamine 3000 was utilized for the transfection of oligonucleotides and recombinant plasmids.

### MTT assay

After culture in 96-well plates for 24 h, HCC cells were transfected with the corresponding oligonucleotides and cultured for another 24 h. Next, 20 μl dye solution was added into each well and incubated at 37 °C for 4 h. Stop solution (200 μl) was then added to stop the reaction, and absorbance was detected using Infinite^®^ 200 PRO (FPRO-T; Tecan, Seestrasse, Switzerland) at 490 nm.

### Colony formation assay

Treated HCC cells (3000 cells/well) were cultured in 6-well plates containing DMEM in a cell incubator with 95% O_2_ and 5% CO_2_. After incubating at 37 °C for two weeks, the cell colonies were fixed and incubated with Giemsa solution for 10 min. The visible colonies were counted under a ×20 microscope.

### Xenograft assay

The eight-week-old BALB/c mice (male) used in this study were provided by The Third Affiliated Hospital, Sun Yat-sen University, and the animal experiments were approved by the Institutional Animal Care and Use Committee of The Third Affiliated Hospital, Sun Yat-sen University. SMMC-7721 cells carrying Lv-sh-NC or Lv-sh-circ_0001955 were collected and suspended in DMEM at 1 × 10^5^ cells/ml. Next, SMMC-7721 cell suspensions (100 μL) were subcutaneously injected into the back of mice randomly and allowed to grow for 25 days. Xenograft volume and weight were examined.

### Plasmid construction and dual luciferase reporter assay

HepG2 cells (1 × 10^4^ cells/well) were plated into 96-well plates overnight and then cotransfected with Luc-circ_0001955, Luc-circ_0001955 mut, Luc-TRAF6 or Luc-MAPK11, and miR-516a-5p or miR-516a-5p mut using Lipofectamine 3000 (Invitrogen) following the manufacturer’s instructions. After 48 h of transfection, firefly and Renilla luciferase activities were determined with the Dual-Luciferase Assay System (Promega).

### Annotations of circRNA/miRNA interactions and KEGG analysis

Putative target miRNAs of circ_0001955 were screened by using starBase (version 3.0) and Circular RNA Interactome. Biological pathways of miR-145-5p and miR-516a-5p were explored using Kyoto Encyclopedia of Genes and Genomes (KEGG; http://www.genome.jp/kegg/).

### CircRNA-miRNA-mRNA coexpression network analysis

The circRNA-miRNA-mRNA coexpression network of circ_0001955 was established with miR-145-5p and miR-516a-5p and their target genes using Cytoscape software (version 3.4.0).

### Biotin pull-down assay

Biotin-miR-NC, Biotin-miR-516a-5p, and Biotin-miR-516a-5p mut, obtained from Integrated Biotech Solutions (Shanghai, China), were individually transfected into HepG2 cells. Forty-eight hours after transfection, HepG2 cells were lysed using lysis buffer, and cell lysate was added to 50 μl beads-containing solution (Dynabeads MyOne Streptavidin C1, Life Technologies). After sufficient mixing, TRIzol Reagent (Life Technologies) was used to extract RNA from the beads, followed by qRT-PCR detection.

### Western blot assay

Treated HepG2 cells were collected and lysed with RIPA buffer containing a protease inhibitor cocktail (Sigma, USA), and the concentration of protein was determined by a BCA kit (Beyotime, China). Extracted proteins were separated in 10% SDS-PAGE and then transferred to PVDF membranes (Millipore, USA) followed by incubation with the following primary antibodies: anti-TRAF6 (rabbit, ab137452, Abcam), anti-MAPK11 (rabbit, ab137066, Abcam), and anti-GAPDH (rabbit, ab181602, Abcam).

### RNA immunoprecipitation

An Ago2-based immunoprecipitation experiment was conducted in HepG2 cells to validate whether circ_0001955 and miR-516a-5p exist in the same RNA-induced silencing complex (RISC). Briefly, HepG2 cells were collected 48 h after miR-NC, miR-516a-5p or miR-516a-5p mut transfection. Treated HepG2 cells were lysed and centrifuged at 12,000 rpm/min for 20 min, and then anti-FLAG M2 magnetic beads (20 μl) were added to the cell lysate. After sufficient mixing and washing three times, the pull-down complexes were analyzed using qRT-PCR.

### Statistical analysis

Data are shown as the mean ± SEM. Two-tailed Student’s *t*-test, performed using GraphPad (Prism ver. 7, GraphPad Prism Software, La Jolla, CA, USA), was utilized to analyze statistical significance between groups, and a *P* value less than 0.05 was considered significant.

## Results

### Circ_0001955 was found to be upregulated in HCC

**Fig. 1:**
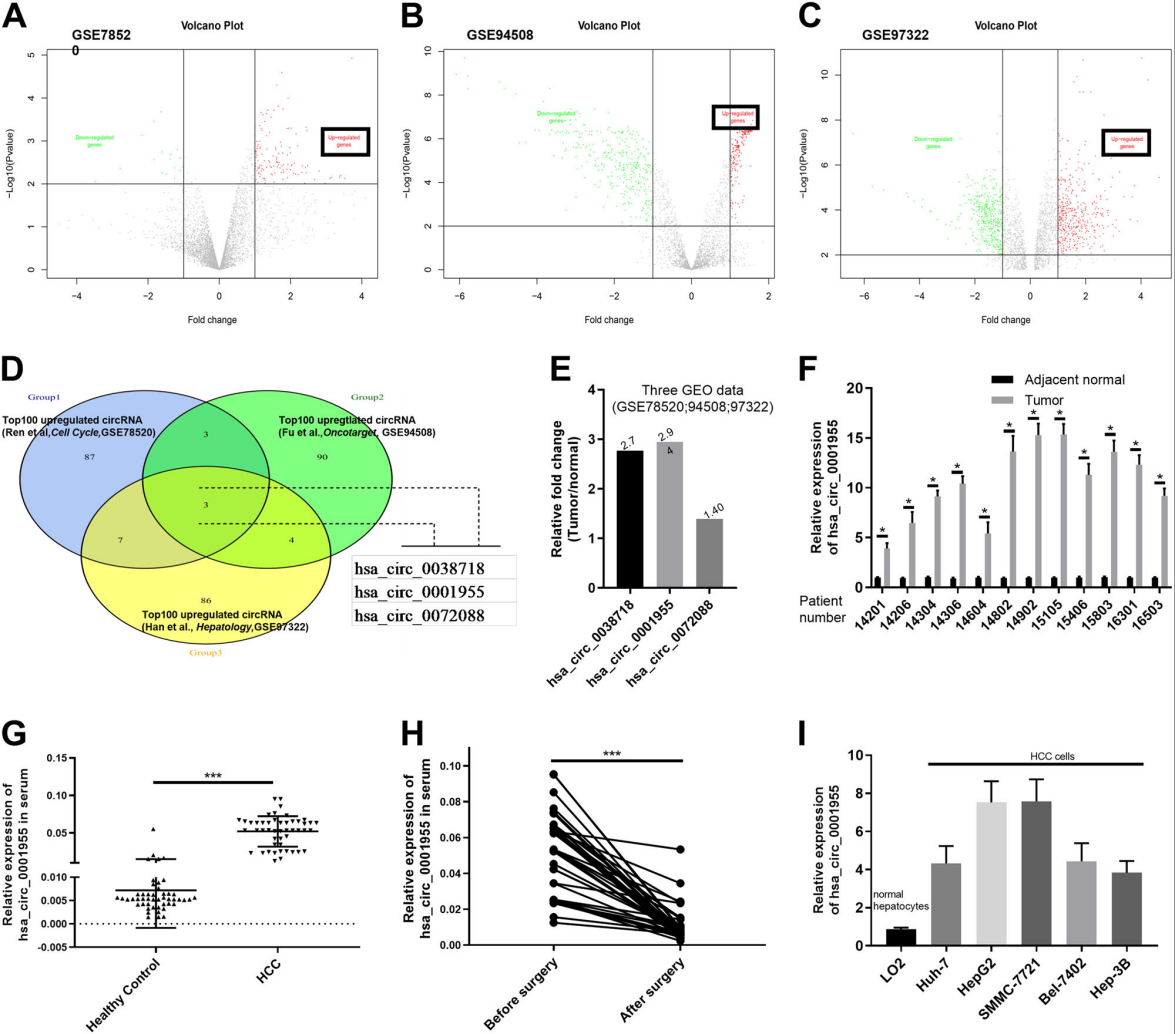
Circ_0001955 was found to be upregulated in HCC. **a**–**c** Volcano plots indicate dysregulated circRNAs between HCC and normal samples from the GSE7852, GSE94508 and GSE97322 datasets. **d** Venn diagram showing the intersection. **e** Relative fold changes of circ_0038718, circ_0001955 and circ_0072088. **f** Relative expression level of circ_0001955 was analyzed by qRT-PCR in tumor and adjacent normal specimens from HCC patients, *P < 0.05. **g** Serum circ_0001955 level was examined by qRT-PCR in healthy control and HCC patients, ***P < 0.001. **h** Serum circ_0001955 level of HCC patients before and after surgery, ***P < 0.001. **i** qRT-PCR analysis of circ_0001955 in the normal hepatocyte LO2 cell line and HCC cell lines (Huh-7, HepG2, SMMC-7721, Bel-7402, and Hep-3B).

To identify unique circRNAs involved in HCC, we analyzed the microarray data of GSE7852, GSE94508, and GSE97322 downloaded from the GEO database and then visualized the differentially expressed circRNAs (DEcircRNAs) in HCC and normal tissue samples by the GEO2R method (Fig. 1a–c). Among the DEcircRNAs, circ_0038718, circ_0001955, and circ_0072088 were the only circRNAs appearing in all three GSE datasets (Fig. 1d), and circ_0001955 exhibited the highest relative fold change (Fig. 1e). Therefore, circ_0001955 was selected for further study. Circ_0001955 is located in the CSNK1G1 gene and is formed by head-to-tail splicing of CSNK1G1 exons 4–9 (Supplemental Fig. 1a). Convergent and divergent primers were designed to amplify circ_0001955 from gDNA and cDNA of HCC tissues. The results showed that circ_0001955 could only be amplified by the divergent primers from cDNA (Supplemental Fig. 1b). RNase R exonuclease was utilized to further validate circ_0001955 in HepG2 and Huh-7 cells. RNase R exonuclease exposure could degrade CSNK1G1 mRNA, while it had no effect on circ_0001955 (Supplemental Fig. 1c). Next, we detected circ_0001955 expression in 12 pairs of HCC and adjacent normal tissue specimens via qRT-PCR. The results indicated that circ_0001955 was increased in HCC samples compared to normal samples (*P* < 0.05, Fig. 1f). Moreover, qRT-PCR examination of circ_0001955 showed that its expression was remarkably higher in the serum of HCC patients than in that of healthy controls (*P* < 0.001, Fig. 1g). After surgery, the serum circ_0001955 expression of HCC patients was significantly reduced (*P* < 0.001, Fig. 1h). We also detected circ_0001955 expression in HCC cell lines by qRT-PCR. Compared to that in the normal hepatic cell line LO2, circ_0001955 was markedly upregulated in Huh-7, HepG2, SMMC-7721, Bel-7402, and Hep-3B cells (Fig. 1i). These findings suggested that increased circ_0001955 may be involved in the tumorigenesis of HCC.

### Circ_0001955 acted as an oncogene in HCC

**Fig. 2:**
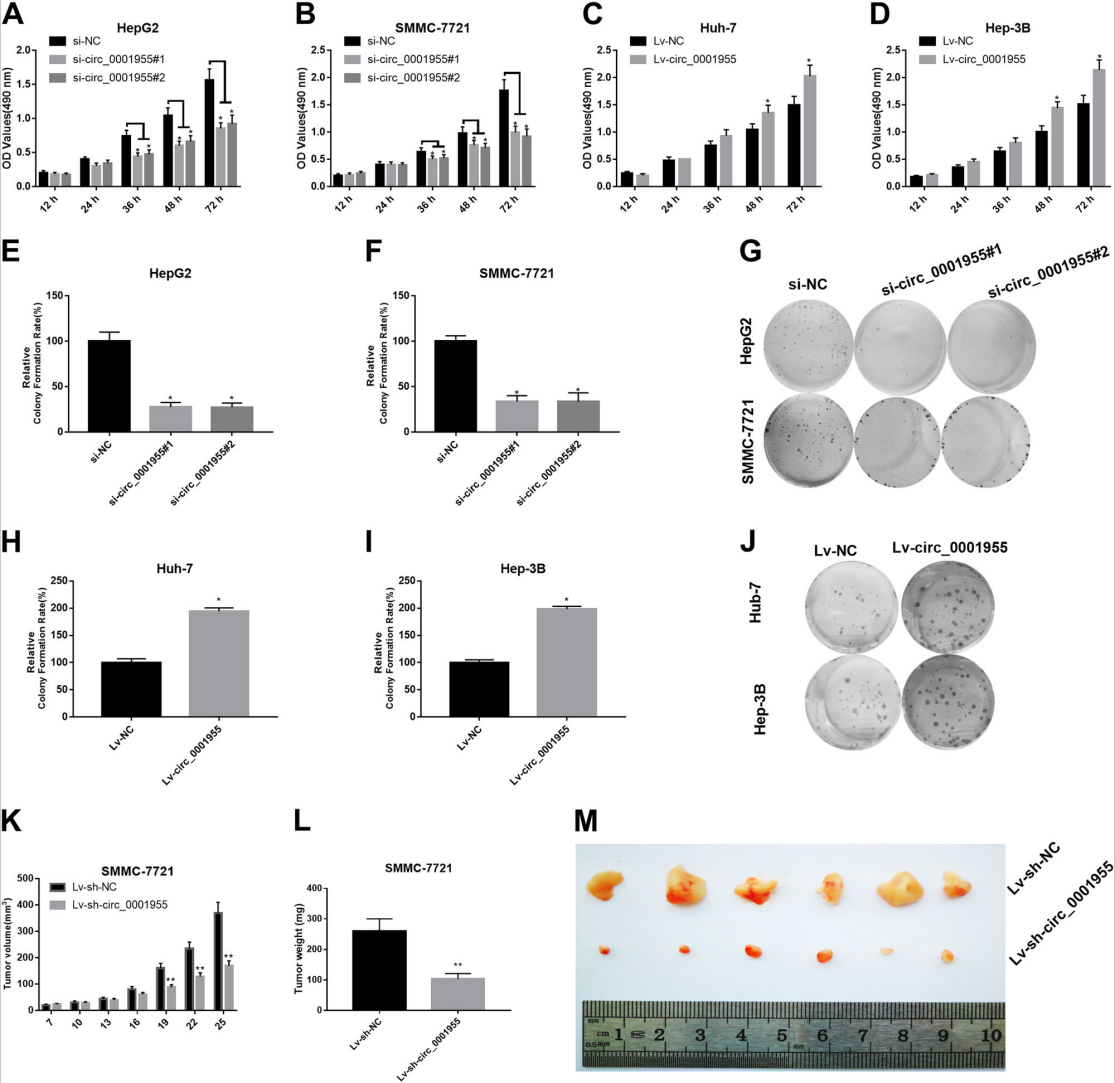
Circ_0001955 acted as an oncogene in HCC. MTT assay was performed in si-circ_0001955#1-transfected and si-circ_0001955#2-transfected (**a**) HepG2 and (**b**) SMMC-7721 cells, as well as in Lv-circ_0001955-transfected (**c**) Huh-7 and (**d**) Hep-3B cells, *P < 0.05. **e**–**g** The effect of circ_0001955 knockdown on HepG2 and SMMC-7721 cells was evaluated by colony formation assay, *P < 0.05. **h**–**j** The effect of circ_0001955 overexpression on Huh-7 and Hep-3B cells was examined by colony formation assay, *P < 0.05. **k**–**m** SMMC-7721 cells stably transfected with Lv-sh-NC and Lv-sh-circ_0001955 were subcutaneously injected into nude mice followed by examination of tumor volume and weight, **P < 0.01.

Subsequently, we detected the effect of circ_0001955 knockdown and overexpression on HCC tumor progression in vitro and in vivo. qRT-PCR was performed in HepG2 cells transfected with circ_0001955 siRNAs (si-circ_0001955#1 and si-circ_0001955#2) and Huh-7 cells transfected with Lv-circ_0001955 to examine the knockdown and overexpression efficiency. Treatment with si-circ_0001955#1 or si-circ_0001955#2 resulted in a significant downregulation of circ_0001955 in HepG2 cells (*P* < 0.05, Supplemental Fig. 2a), and Lv-circ_0001955 treatment caused a remarkable upregulation of circ_0001955 in Hun-7 cells (*P* < 0.05, Supplemental Fig. 2b). The MTT assay performed in HCC cells demonstrated that circ_0001955 knockdown remarkably attenuated the proliferation of HepG2 and SMMC-7721 cells (*P* < 0.05, Fig. 2a, b), whereas circ_0001955 overexpression enhanced the proliferation of Huh-7 and Hep-3B cells (*P* < 0.05, Fig. 2c, d). A colony formation assay was subsequently performed in vitro to examine the effect of circ_0001955 on the clonogenic capacity of HCC cells. The results indicated that circ_0001955 knockdown led to a remarkable downregulation of the colony number of HepG2 and SMMC-7721 cells (*P* < 0.05, Fig. 2e–g), whereas circ_0001955 overexpression had the opposite effect on Huh-7 and Hep-3B cells (*P* < 0.05, Fig. 2h–j). These results indicated that circ_0001955 promoted HCC cell proliferation in vitro. To further confirm its oncogenic role in HCC progression, an in vivo tumor growth assay was carried out. Circ_0001955-silenced SMMC-7721 cells were subcutaneously injected into male nude mice, and then tumor volume and weight were examined in the following 25 days. Tumors derived from the circ_0001955-silenced SMMC-7721 cells were obviously smaller and lighter than those of the control groups (*P* < 0.01, Fig. 2k–m).

### MiR-516a-5p directly bound circ_0001955 in HCC

**Fig. 3:**
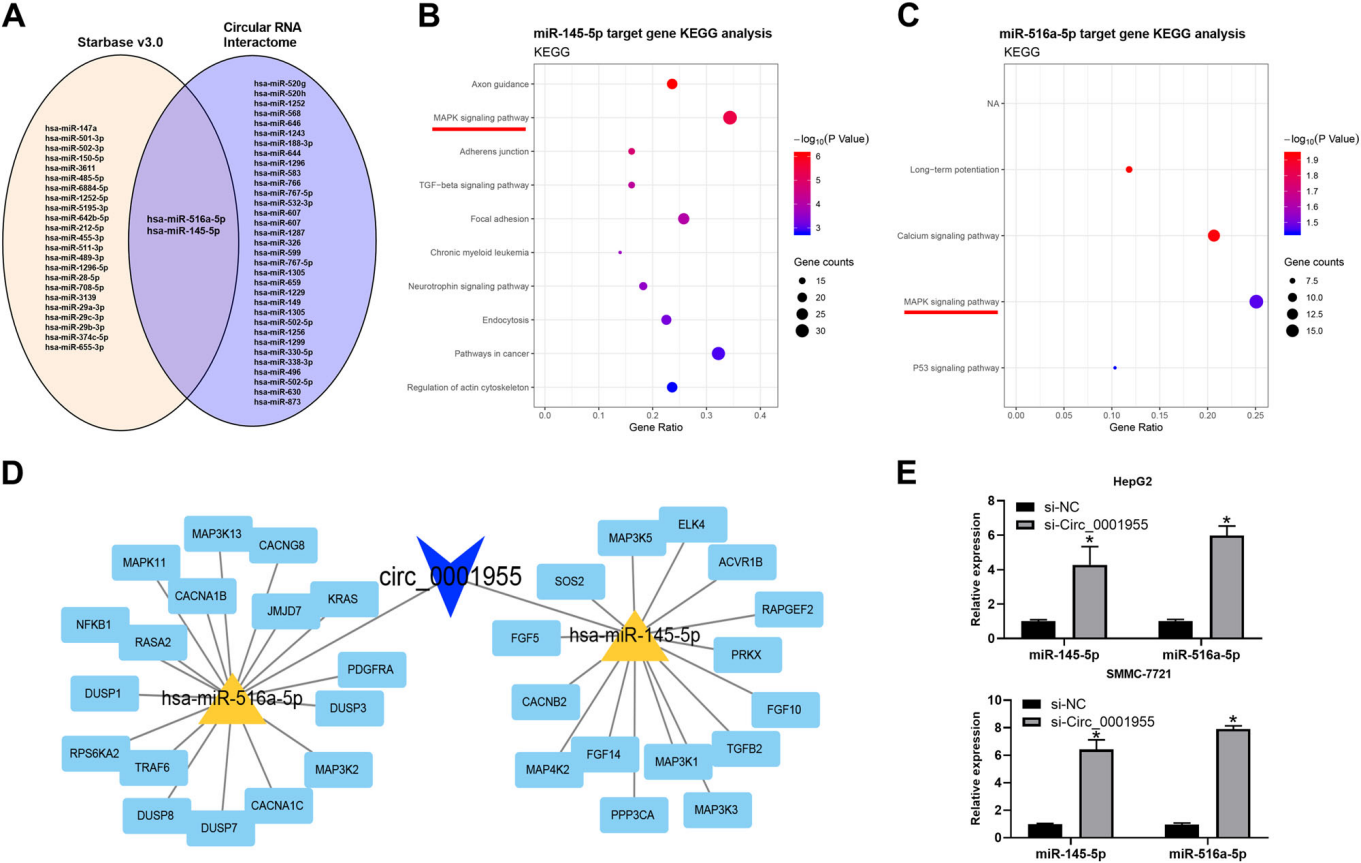
Identification of target miRNAs of circ_0001955. **a** Two bioinformatics analysis software programs (starBase and Circular RNA Interactome) were utilized to predict the target miRNAs of circ_0001955. **b, c** KEGG pathway analysis of miR-145-5p and miR-516a-5p. **d, e** The ceRNA network of circ_0001955 with miR-145-5p and miR-516a-5p and their target genes. **e** Relative expression of miR-145-5p and miR-516a-5p was measured in HepG2 and SMMC-7721 cells treated with si-NC and si-circ_0001955 using qRT-PCR, *P < 0.05.

**Fig. 4:**
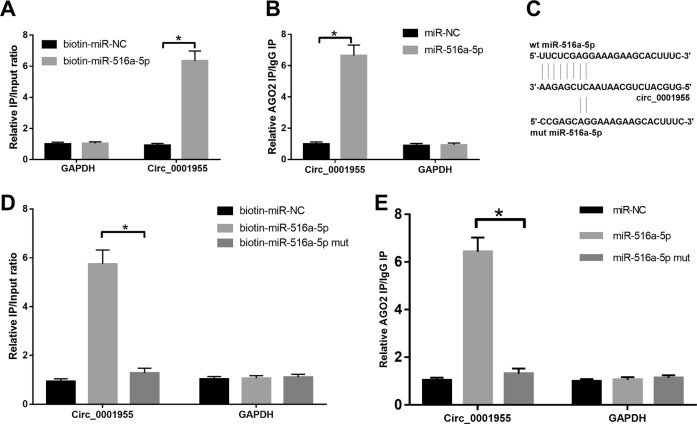
miR-516a-5p was targeted by circ_0001955. **a** A biotin-miRNA assay was utilized to verify the interaction between miR-516a-5p and circ_0001955, *P < 0.05. **b** Immunoprecipitation of AGO2 was performed in HCC cells to examine the interaction between miR-516a-5p and circ_0001955, *P < 0.05. **c** The sequence of wild-type (wt) and mutant (mut) miR-516a-5p. **d** Biotin-labeled miR-516a-5p (biotin-miR-516a-5p) and its negative control (biotin-miR-516a-5p mut) were transfected into HCC cells, and the streptavidin-captured circ_0001955 and GAPDH were detected by qRT-PCR, *P < 0.05. **e** AGO2 was cotransfected into HCC cells with miR-NC, miR-516a-5p and miR-516a-5p mut followed by detection of circ_0001955 and GAPDH, *P < 0.05.

To understand the mechanism of circ_0001955 in HCC tumorigenesis, we screened the potential targets of circ_0001955 by starBase (version 3.0) and Circular RNA Interactome, and the predicted results are shown in a Venn diagram. Only two miRNAs (miR-516a-5p and miR-145-5p) appeared in both the starBase and Circular RNA Interactome (Fig. 3a). We predicted the target genes of miR-516a-5p and miR-145-5p by TargetScan7.2 and subsequently performed KEGG pathway analysis on the identified target genes. The top ten pathways of miR-516a-5p and miR-145-5p are shown, and the MAPK signaling pathway was enriched in both the miR-516a-5p and miR-145-5p KEGG analysis (Fig. 3b, c). A competing endogenous RNA (ceRNA) network showed the molecular interaction of circ_0001955 with miR-516a-5p and miR-145-5p and their target genes (Fig. 3d). Moreover, by using qRT-PCR, we found that the relative expression of miR-516a-5p and miR-145-5p was remarkably increased in circ_0001955-silenced HepG2 and SMMC-7721 cells (*P* < 0.05, Fig. 3e). An RNA pull-down assay was performed to validate the interplay between circ_0001955 and miR-516a-5p. The results indicated that circ_0001955 could be pulled down by biotinylated miR-516a-5p (biotin-miR-516a-5p) but not by biotinylated scramble negative control (biotin-miR-NC) (*P* < 0.05, Fig. 4a). When the circ_0001955-targeted sequence of miR-516a-5p was mutated, no circ_0001955 was detected following affinity purification, implying that circ_0001955 interacted with miR-516a-5p in a sequence-specific manner (*P* < 0.05, Fig. 4c, d). Because the regulatory effects of miRNAs on gene expression are dependent on the RNA-induced silencing complex (RISC), which contains Ago2 protein, we then adopted an RNA-binding protein immunoprecipitation assay to examine whether miR-516a-5p and circ_0001955 form a RISC. Circ_0001955 was enriched in the Ago2 complex (*P* < 0.05, Fig. 4b), which could be abrogated when the circ_0001955-targeted sequence of miR-516a-5p was mutated (*P* < 0.05, Fig. 4e). Overall, miR-516a-5p bound directly to circ_0001955 in HCC cells.

### MiR-516a-5p target genes were modulated by circ_0001955 in HCC cells

**Fig. 5:**
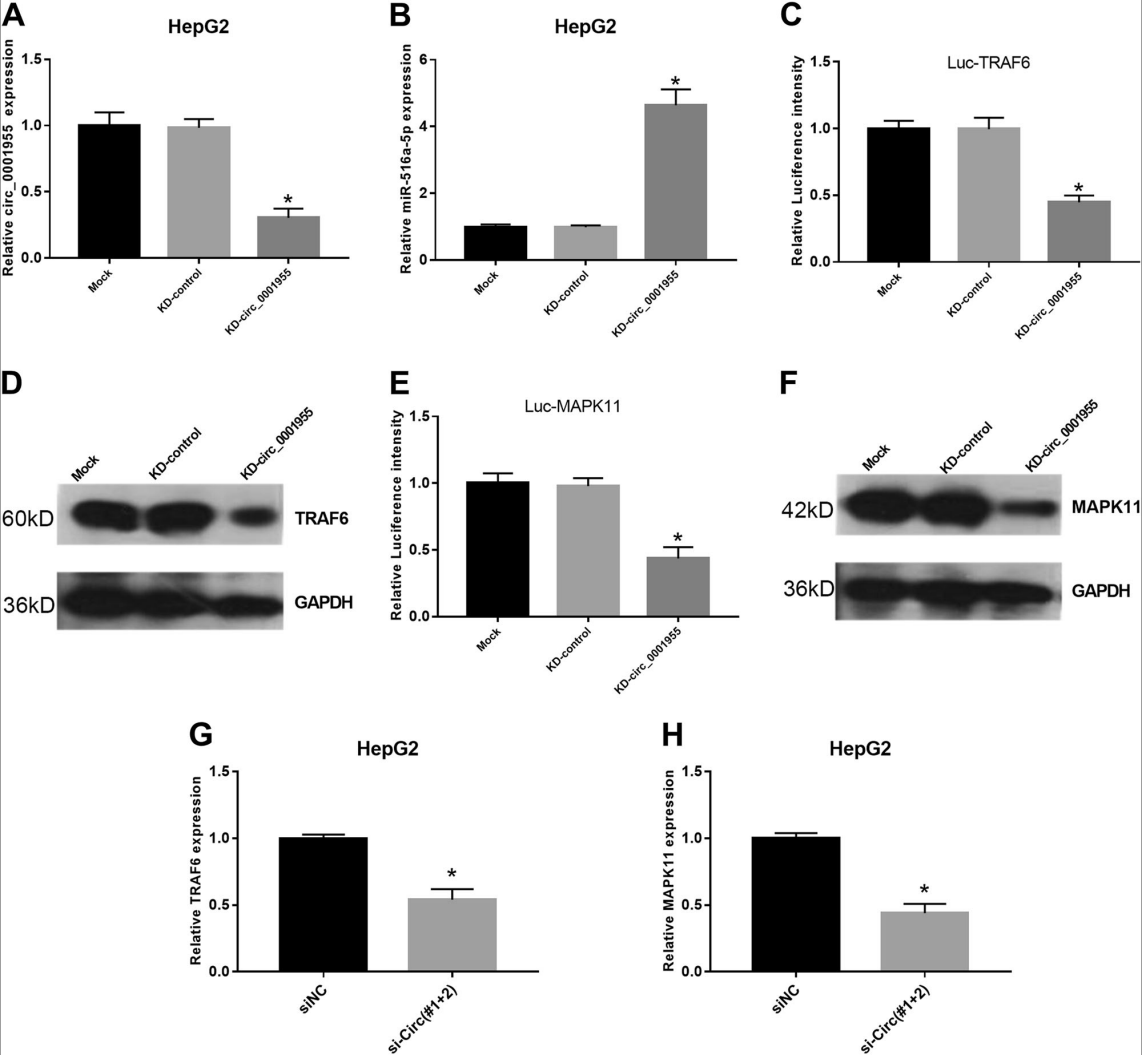
MiR-516a-5p target genes were modulated by circ_0001955 in HCC cells. **a** The expression levels of circ_0001955 and (**b**) miR-516a-5p were detected by qRT-PCR in HepG2 cells transfected with control siRNA or circ_0001955 siRNAs, *P < 0.05. After transfection with control siRNA or circ_0001955 siRNAs, the luciferase activity of HCC cells driven by (**c**) Luc TRAF6 or (**e**) Luc MAPK11 was analyzed. After transfection with control siRNA or circ_0001955 siRNAs, the protein expression levels of (**d**) TRAF6 and (**f**) MAPK11 were measured via western blot, and the mRNA expression levels of (**g**) TRAF6 and (**h**) MAPK11 were examined by qRT-PCR, *P < 0.05.

KEGG pathway analysis implicated multiple target genes of miR-516a-5p, including TRAF6 and MAPK11. Considering the interaction between circ_0001955 and miR-516a-5p in HCC, we wondered whether TRAF6 and MAPK11 could be regulated by circ_0001955. We transfected HepG2 cells with si-circ_0001955#1 and si-circ_0001955#2 to knockdown circ_0001955 (KD-circ_0001955) (*P* < 0.05, Fig. 5a). Silencing of circ_0001955 remarkably upregulated miR-516a-5p levels in HepG2 cells (*P* < 0.05, Fig. 5b). In the luciferase reporter assay, we found that knockdown of circ_0001955 repressed the luciferase activity of HepG2 cells driven by Luc-TRAF6 (*P* < 0.05, Fig. 5c) and Luc-MAPK11 (*P* < 0.05, Fig. 5e). In addition, circ_0001955 knockdown was demonstrated to reduce the expression of TRAF6 (*P* < 0.05, Fig. 5d, g) and MAPK11 (*P* < 0.05, Fig. 5f, h) at both the protein and mRNA levels. Thus, circ_0001955 could indirectly regulate the miR-516a-5p target genes TRAF6 and MAPK11.

### Circ_0001955 functioned as a miR-516a-5p sponge in HCC cells

**Fig. 6:**
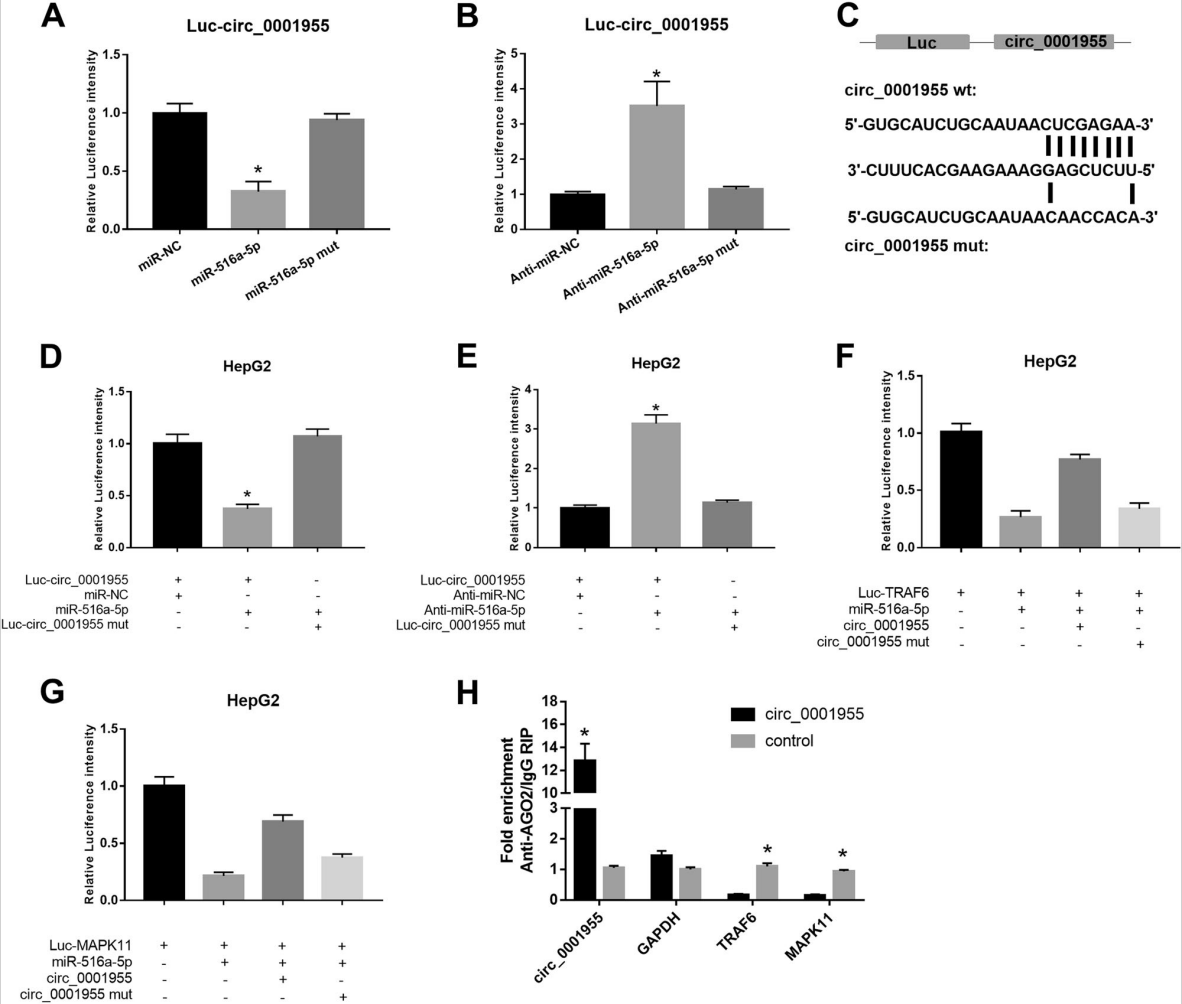
Circ_0001955 functioned as a miR-516a-5p sponge in HCC cells. **a, b** Luc-circ_0001955-driven luciferase activity was evaluated after transfection with miR-516a-5p or anti-miR-516a-5p, *P < 0.05. **c** The sequence of circ_0001955 containing the wild-type or mutant miR-516a-5p binding site. **d**, **e** Luciferase activity of HCC cells cotransfected with circ_0001955-WT or circ_0001955-mut and miR-516a-5p or anti-miR-516a-5p were determined, *P < 0.05. **f, g** Rescue assay showing the effects of circ_0001955 on the interaction between miR-516a-5p and TRAF6 or MAPK11. **h** Ago-associated immunoprecipitation was performed in HepG2 cells transfected with control or circ_0001955 vector, followed by the detection of circ_0001955, TRAF6 and MAPK11 expression levels using qRT-PCR, *P < 0.05.

A dual-luciferase reporter assay was conducted in HCC cells to investigate whether miR-516a-5p is targeted by circ_0001955 using specific miR-516a-5p constructs containing wild-type (WT) and mutant (mut) circ_0001955 binding sites. The results indicated that miR-516a-5p transfection decreased the luciferase activity of HCC cells driven by Luc-circ_0001955, whereas no effects were observed in the miR-516a-5p mut-transfected HCC cells (*P* < 0.05, Fig. 6a). Moreover, HCC cells transfected with anti-miR-516a-5p but not anti-miR-516a-5p mut enhanced the luciferase activity of HCC cells driven by Luc-circ_0001955 (*P* < 0.05, Fig. 6b). Moreover, when we mutated the sequence of WT circ_0001955 into circ_0001955 mut (Fig. 6c), miR-516a-5p and anti-miR-516a-5p could no longer affect the luciferase activity (*P* < 0.05, Fig. 6d, e), indicating that only WT circ_0001955 could be recognized by miR-516a-5p and anti-miR-516a-5p. In addition, we found that cotransfection of miR-516a-5p and Luc-TRAF6 or Luc-MAPK11 suppressed the luciferase activity of HepG2 cells; however, circ_0001955 treatment restored the reduction in luciferase activity (Fig. 6f, g). In addition, we revealed that circ_0001955 overexpression markedly increased the enrichment of Ago2 on circ_0001955, whereas it substantially reduced the enrichment on TRAF6 and MAPK11 (*P* < 0.05, Fig. 6h). These data suggest that circ-0001955 indirectly regulated the miR-516a-5p-targeted genes TRAF6 and MAPK11 by functioning as a miR-516a-5p sponge.

### The correlation between miR-516a-5p, TRAF6 or MAPK11 and circ_0001955 in HCC

**Fig. 7:**
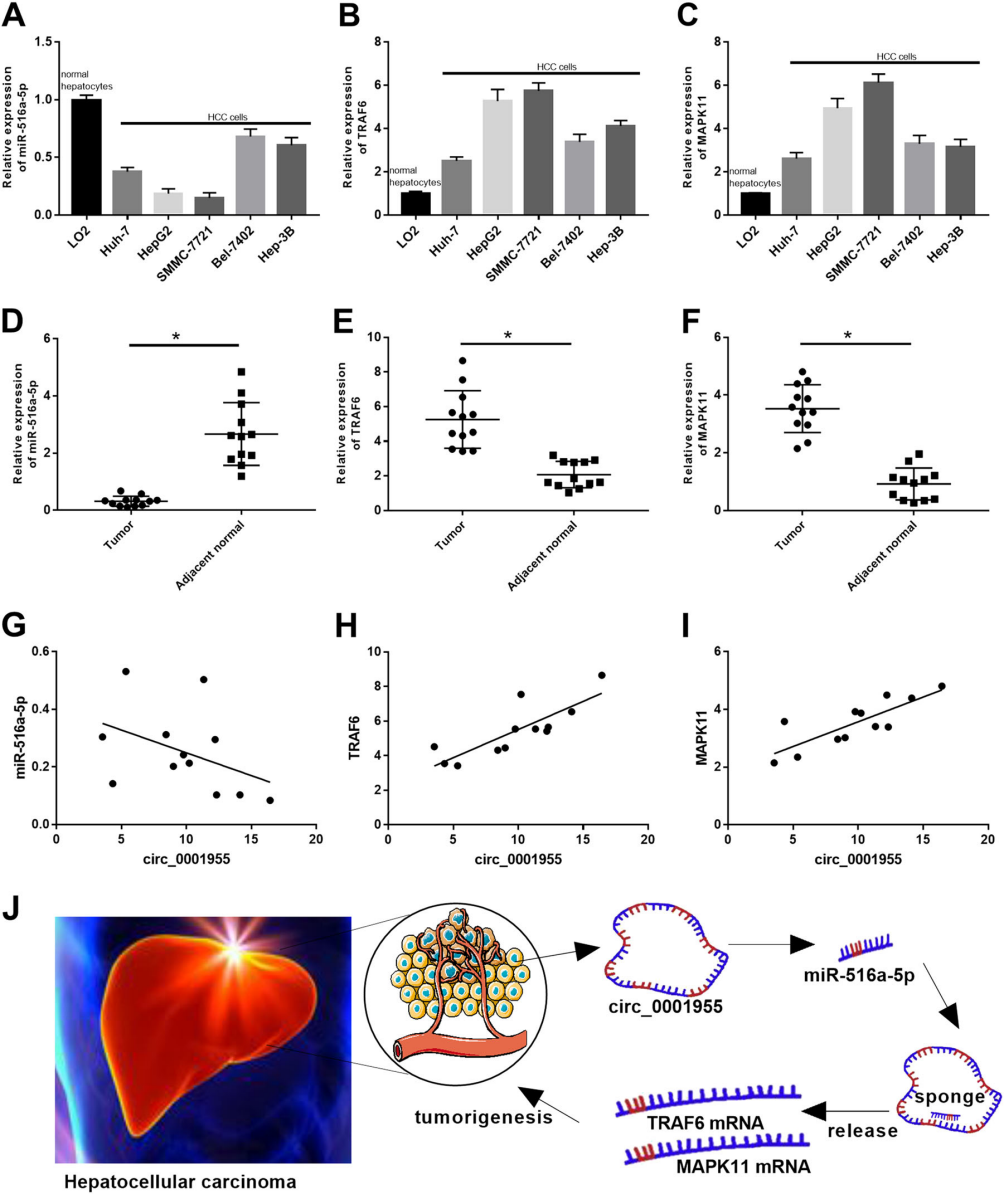
The correlation between miR-516a-5p, TRAF6 or MAPK11 and circ_0001955 in HCC. Relative expression of (**a**) miR-516a-5p, (**b**) TRAF6, and (**c**) MAPK11 in HCC cell lines was examined by qRT-PCR. qRT-PCR analysis of (**d**) miR-516a-5p, (**e**) TRAF6, and (**f**) MAPK11 in HCC tumor samples and adjacent nontumor samples, *P < 0.05. Pearson correlation analysis of (**g**) miR-516a, (**h**) TRAF6, or (**i**) MAPK11 expression and circ_0001955 expression in HCC samples. **j** The diagram of the mechanisms underlying the circ_0001955/miR-516a-5p axis in HCC.

The expression of miR-516a-5p, TRAF6 and MAPK11 in HCC cell lines was assessed via qRT-PCR. Compared to that in LO2 cells, miR-516a-5p expression in Huh-7, HepG2, SMMC-7721, Bel-7402, and Hep-3B cells was obviously decreased (Fig. 7a), while TRAF6 and MAPK11 expression levels in Huh-7, HepG2, SMMC-7721, Bel-7402, and Hep-3B cells were significantly increased (Fig. 7b, c). Moreover, the downregulation of miR-516a-5p and upregulation of TRAF6 and MAPK11 were confirmed by qRT-PCR in HCC tumor samples (*P* < 0.05, Fig. 7d–f). In addition, the results from Pearson correlation analysis in 12 HCC samples showed that miR-516a-5p expression was negatively correlated with circ_0001955 expression (Fig. 7g), while TRAF6 and MAPK11 expression was positively correlated with circ_0001955 expression (Fig. 7h, i). Taken together, our findings indicated that circ_0001955 promoted HCC tumorigenesis by sponging miR-516a-5p to release TRAF6 and MAPK11 expression (Fig. 7j).

## Discussion

Due to the high potential of metastasis, HCC has long been an aggressive malignant cancer, increasingly threatening people’s health^19^. Searching for target molecules involved in the pathogenesis of HCC has long been considered an effective research strategy for conquering this disease^20,21^. However, the efforts of several generations did not yield much due to technological limitations. In recent decades, genomics studies have made tremendous progress due to the wide application of next-generation sequencing technology. It is well known that noncoding RNAs account for more than 98% of the genome, with only 2% encoding protein^22^. As an important subtype of ncRNAs, circRNAs have been revealed to act as oncogenes or tumor suppressors in HCC through multiple mechanisms^13^. For instance, Wang Z et al. reported that circHIAT1 overexpression repressed HCC cell growth in vitro by regulating the PTEN pathway through miR-3171^23^. Recently, circARSP91 was demonstrated to increase the cytotoxicity of natural killer cells against HCC by upregulating the expression of UL16 binding protein 1^24^. Moreover, another study revealed that circ_ZEB1.33 enhanced the proliferation of HCC cells in vitro by sponging miR-200a-3p to release CDK6^25^. These findings imply that circRNAs can be used as potential therapeutic RNA molecules for HCC. Many circRNAs have been identified as having significant potential for the diagnosis of HCC in the clinic^26^. Nevertheless, the regulatory network of circRNAs in HCC remains largely unclear.

Here, circ_0001955, derived from the CSNK1G1 gene exons 4–9, was identified as a novel HCC-related circRNA in three GSE datasets by using the GEO2R online tool. qRT-PCR validation showed that circ_0001955 was markedly increased in HCC, and functional assays provided strong evidence that circ_0001955 acted as an oncogene of HCC in vitro and in vivo. Our findings not only contributed to improve the circRNA regulatory network in HCC but also provided a novel potential therapeutic target for treating HCC patients. Furthermore, in this mechanism, we showed that the promoting effects of circ_0001955 were indirectly mediated by the miR-516a-5p-targeted genes TRAF6 and MAPK11.

Of note, the circRNA-miRNA-mRNA axis has been well documented by numerous studies to control tumor growth at both the transcriptional and posttranscriptional levels^27,28^. We therefore screened the miRNAs that contained complementary sequences of circ_0001955 through two bioinformatics software programs, starBase and Circular RNA Interactome, followed by a KEGG pathway analysis. Two miRNAs (miR-145-5p and miR-516a-5p) were predicted to be the target miRNAs of the circRNA by both starBase and Circular RNA Interactome. Moreover, KEGG pathway analysis demonstrated that the potential downstream genes of both axes circ_0001955/miR-145-5p and circ_0001955/miR-516a-5p correlated with the MAPK signaling pathway. The MAPK signaling pathway has been reported to be involved in the tumorigenesis of HCC by several studies. For example, the MAPK signaling pathway was revealed to mediate an inhibitory effects of miR-148a-3p knockdown on HCC progression^29^. Moreover, decreased long noncoding RNA H19 was reported to induce oxidative stress and attenuate the chemotherapy resistance of HCC cells by suppressing the MAPK signaling pathway^30^. Here, our findings suggest that TRAF6 and MAPK11 (two miR-516a-5p target genes) were significantly upregulated in HCC cells, and they could be indirectly regulated by circ_0001955 in HCC cells. In conclusion, we provide strong evidence that circ_0001955 facilitates HCC tumorigenesis by sponging miR-516a-5p to release TRAF6 and MAPK11.

## Supplementary information

Supplementary Figure Legends

Supplementary Figure 1

Supplementary Figure 2
