# Supplementary material for: Circ_0001955 facilitates hepatocellular carcinoma (HCC) tumorigenesis by sponging miR-516a-5p to release TRAF6 and MAPK11
Source: Cell Death Dis. 2019 Dec 10;10(12):945. doi: 10.1038/s41419-019-2176-y (PMC6904727; doi:10.1038/s41419-019-2176-y)
Supplement: Supplementary file 1 — Supplementary Figure Legends [file 41419_2019_2176_MOESM1_ESM.docx]

**Figure S1. Validation of circ_0001955. (A)** The location of circ_0001955 in its parental genome CSNK1G1. **(B)** The schematic of circ_0001955 formation, and the validation of circ_0001955 with the cDNA and gDNA from tumor cells. **(C)** qRT-PCR analysis of CSNK1G1 mRNA and circ_0001955 in HepG2 and Huh-7 cells treated with RNase R.

**Figure S2. Validation of circ_0001955 knockdown and overexpression. (A)** Relative expression of circ_0001955 was examined in HepG2 cells transfected with si-NC, si-circ_0001955#1 and si-circ_0001955#2, **P*<0.05. **(B)** qRT-PCR detection of circ_0001955 in Huh-7 cells treated with Lv-NC and Lv-circ_0001955, **P*<0.05.
