# Supplementary figures and images for: Circ_0001955 facilitates hepatocellular carcinoma (HCC) tumorigenesis by sponging miR-516a-5p to release TRAF6 and MAPK11
Source: Cell Death Dis. 2019 Dec 10;10(12):945. doi: 10.1038/s41419-019-2176-y (PMC6904727; doi:10.1038/s41419-019-2176-y)

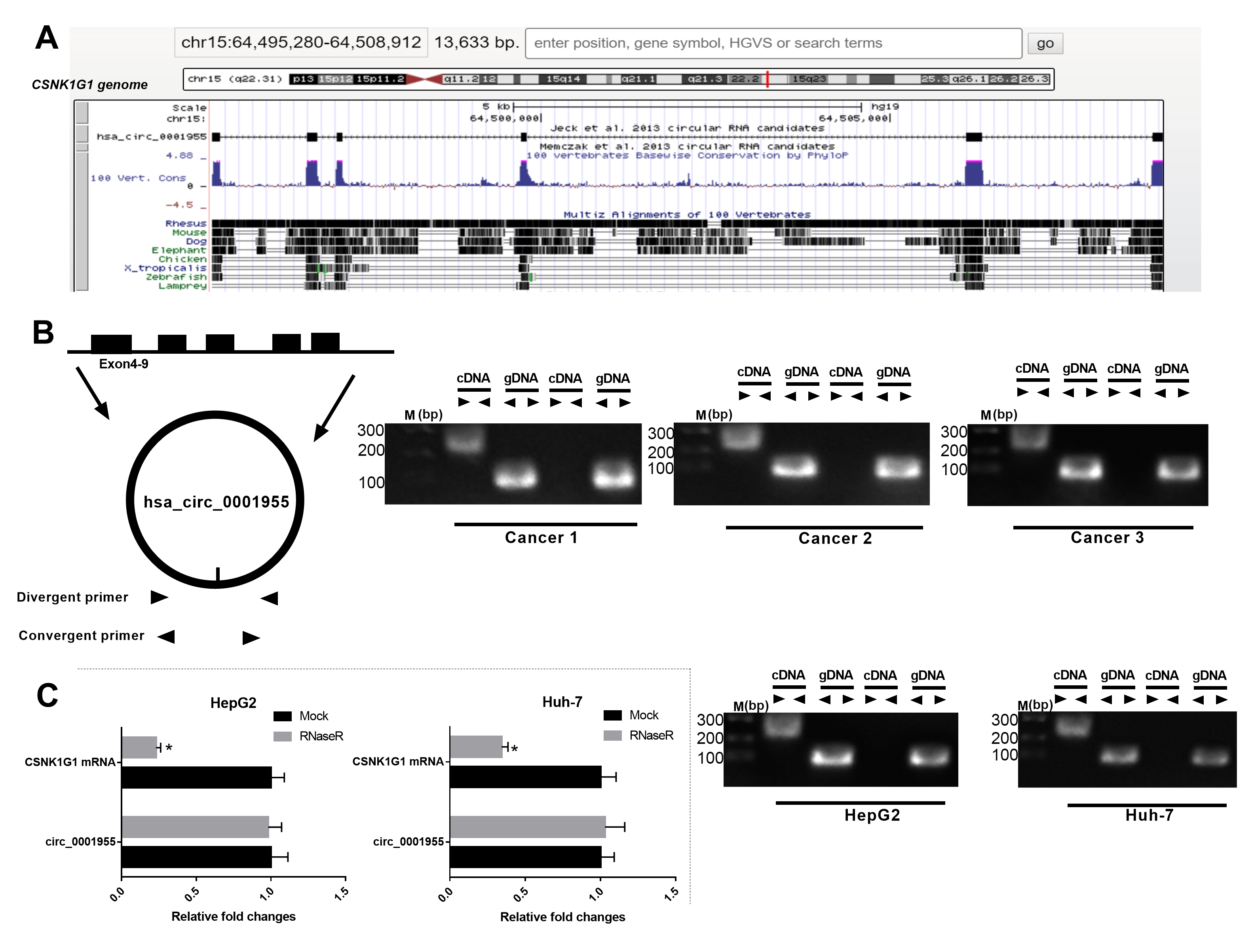

Supplement: Supplementary file 2 — Supplementary Figure 1 [file 41419_2019_2176_MOESM2_ESM.tif]

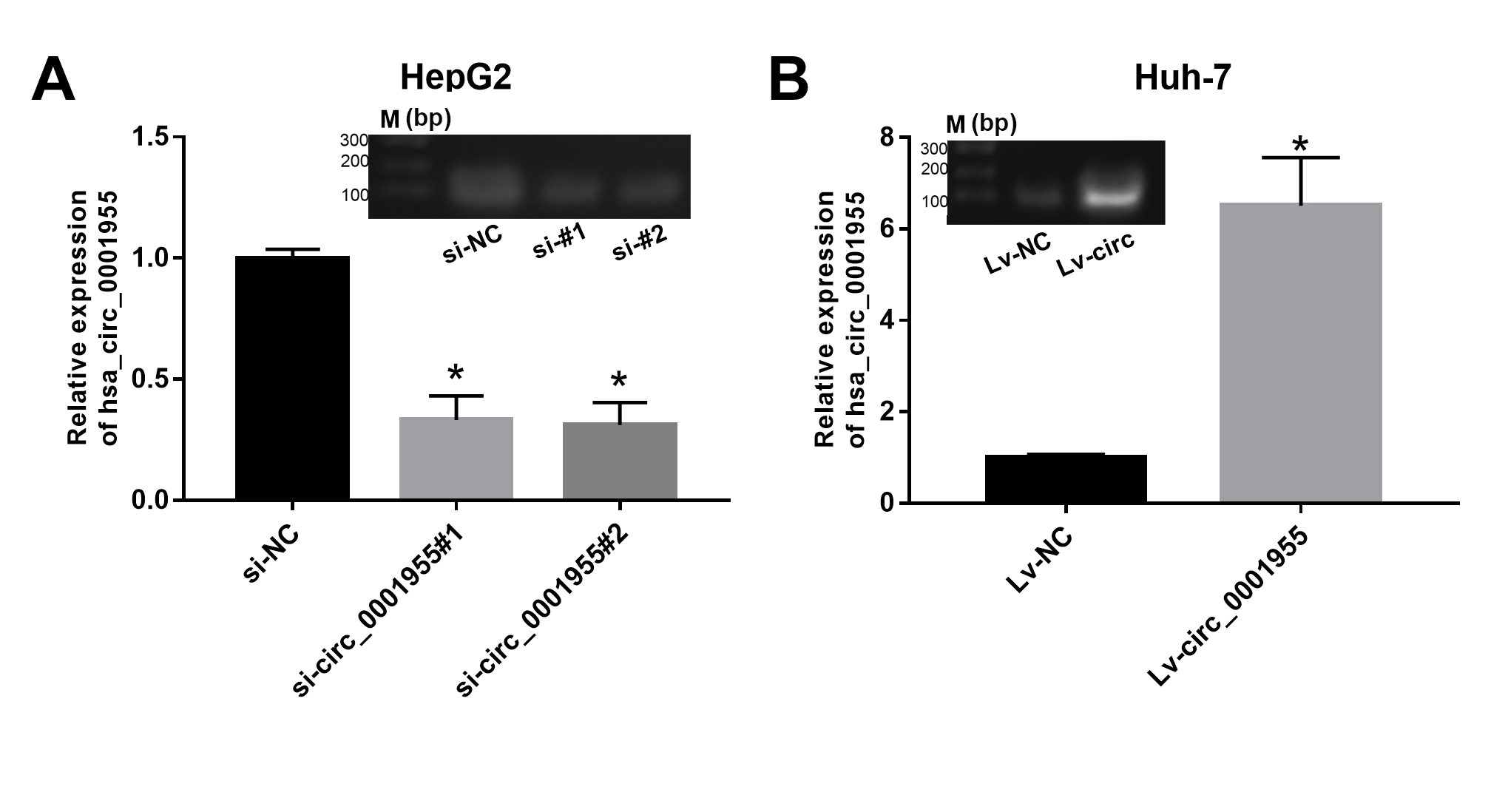

Supplement: Supplementary file 3 — Supplementary Figure 2 [file 41419_2019_2176_MOESM3_ESM.tif]
